# Population groups in dietary transition

**DOI:** 10.3402/fnr.v57i0.21668

**Published:** 2013-10-03

**Authors:** Per E. Wändell

**Affiliations:** Centre for Family Medicine, Karolinska Institutet, Huddinge, Sweden

**Keywords:** Ethnic groups, obesity, diabetes, cardiovascular diseases, vitamin D deficiency, dental caries

## Abstract

**Background:**

Little is known about the effects of dietary acculturation in minority groups in the Nordic countries, including immigrants from non-Western societies.

**Methods:**

A search was performed in Medlin33e/PubMed and SweMed+ for articles published in 1990–2011.

**Results:**

A total of 840 articles were identified, with a final 32 articles used to tabulate results which were included in the primary analysis. High rates of vitamin D deficiency (23 articles) were found in immigrants of non-Western origin; deficiency rates were very high among both pregnant and non-pregnant women, and also among children, with young children of immigrant parents showing 50 times higher risk for rickets when compared to children of indigenous parents. The risk of iron deficiency (two articles) was high among immigrant women, while the results were inconclusive regarding children. High rates of dental caries (seven articles) were found among pre-school and younger school children of immigrant origin, while the risk of caries was not as evident among older children. In a secondary analysis, including 48 articles (results not tabulated), overweight and obesity (14 articles) were seen in many immigrant groups, resulting in a high prevalence of diabetes (2 review articles from a total of 14 original articles) and incidence of coronary heart disease (CHD; seven articles). For hypertension (three articles), dyslipidemia (four articles), and dietary patterns among immigrants (10 articles), the results were contradictory.

**Conclusions:**

Risk of vitamin D deficiency is alarmingly high in the Nordic countries among immigrants of non-Western origin, especially among women. Dental caries is high among immigrant children aged 0–7 years due to a higher intake of sugary products. Overweight and obesity, associated with a higher risk of diabetes and CHD, are prevalent in many immigrant groups and need further attention.

Dietary transition in minority populations could refer to both migrants arriving and acculturating in a new country, and to indigenous ethnic minority groups in a society. Dietary habits in migrant populations of Western countries in Europe, North America, and Australia are likely to become less healthy because of an increased exposure to Western-style fast foods (1). However, ‘it appears that a limited number of foods predict diet quality and health outcomes in various population groups; in particular, fruit and vegetables, fish, wholegrain cereal, and legumes do so on the protective side, and sweets, processed meats, fried foods, fats and oils, and salty snacks do so on the negative side’ (2).

In a literature review of dietary acculturation in the United States, it was concluded that because of the lack of consistency in study designs and findings, it was not possible to draw conclusions about the effects of dietary acculturation on overall diet quality, immigrant-associated dietary patterns, and chronic disease risk (3). The term ‘dietary acculturation’ is defined in that article as the process when members of a minority group in the society adopt the eating patterns of the host country (3). In addition, the concept ‘nutrition transition’ refers to changes in Western societies during the past decades, resulting in more calorie-dense diets and a reduction in physical activity (4).

The topic of acculturation per se could be viewed in different ways, as the process of adaptation to a mainstream culture while giving up or also maintaining the original one (5). The one-dimensional model postulates that the minority and the mainstream cultural identifications have a strong inverse relation. According to the bi-dimensional approach, the acculturation process can result in four directions, becoming 1) integrated – acculturated, yet with preserved ethnic identity; 2) assimilated – acculturated into the mainstream culture at the expense of ethnic identity; 3) separated – not acculturated with preserved ethnic identity; or 4) marginalized – with no strong acculturative pattern or ethnic identity (6). In the ideal situation, immigrants become integrated, while preserving good nutritional habits from their country of origin and at the same time acquiring good nutritional habits from their new home country. However, as stated in one American article, ‘the longer you stay, the bigger you get’ (7), that is, overweight and obesity is a common problem associated with migration. The health problems related to this are described in earlier migration studies, that is, hypertension (8), diabetes (9), and cardiovascular diseases (CVDs) (10, 11).

Reports indicate that Nordic populations generally have more satisfactory vitamin D status compared to populations in southern Europe, likely due to a high consumption of fatty fish and vitamin D supplements, for example, as cod liver oil (12, 13). However, less is known about the effects of the dietary acculturation in minority groups migrating to the Nordic countries, including immigrants from non-Western societies. A review stated that immigrants from the third world are more prone to acquire nutritional deficiency diseases, such as rickets, osteomalacia, and iron deficiency anemia, than the rest of the population in the recipient countries (14). Thus, a focus on some specific dietary inadequacies, such as vitamin D and iron deficiencies, may be needed. Also, there are signs of an increase of diseases due to over-nutrition (14), such as coronary heart disease (CHD) and type 2 diabetes (15) among third world immigrants. In addition, previous reports indicate higher rates of dental caries among immigrants’ children (16), possibly due to more frequent intakes of sweets and sugary beverages. The aim of this paper was to review articles relevant in relation to possible conditions as regards both over-nutrition and connected disorders, and nutritional deficiencies, especially in the most common non-European immigrant groups in Nordic countries.

## Methods

A search was performed in Medline/PubMed and SweMed+. The search terms included the different Nordic countries, immigrants, and ethnic groups, also from specified areas of the world, and different diseases/disorders, risk factors for diseases and specific micronutrient deficiencies (see specific search string!). The time period was limited to articles published in 1990–2011, with studies performed before 1990 excluded.

Population statistics in the Nordic countries was reviewed, with a focus on the most common non-European immigrant groups Table 1.

**Table 1:** Population in the Nordic countries 2010, with regard to countries of origin

	Denmark	Faroe islands	Finland (including Åland)	Iceland	Norway	Sweden
Population	5,534,738	48,568	5,379,161	317,630	4,858,199	9,340,682
All foreign-born (% of all)	501,511 (9.96%)	5,912 (12.17%)	236,766 (4.60%)	35,121 (11.06%)	526,799 (10.84%)	1,337,965 (14.32%)
Nordic countries	76,286 (15.21%)	3,793 (64.16%)	35,543 (15.01%)	6,314 (17.98%)	76,974 (14.61%)	266,519 (19.92%)
Europe (except Nordic c.)	180,532 (36.00%)	492 (8.32%)	114,852 (48.51%)	20,361 (57.97%)	189,672 (36.00%)	441,954 (33.03%)
America	27,909 (5.56%)	147 (2.49%)	9,946 (4.20%)	2,877 (8.19%)	41,650 (7.91%)	92,610 (6.92%)
Africa	35,377 (7.05%)	101 (1.71%)	20,489 (8.65%)	780 (2.22%)	53,579 (10.17%)	103,077 (7.70%)
Western Asia	79,587 (15.87%)	6 (0.10%)	15,992 (6.75%)	193 (0.55%)	47,273 (8.97%)	261,954 (19.58%)
South Asia	27,288 (5.44%)	60 (1.01%)	6,659 (2.81%)	460 (1.31%)	34,932 (6.63%)	37,769 (2.82%)
Southeast Asia	28,705 (5.72%)	133 (2.25%)	12,231 (5.17%)	2,949 (8.40%)	39,505 (7.50%)	48,589 (3.63%)
East Asia	12,012 (2.40%)	18 (0.30%)	7,701 (3.25%)	569 (1.62%)	10,055 (1.91%)	24,096 (1.80%)
Rest of Asia	29,609 (5.90%)	69 (1.17%)	7,389 (3.12%)	454 (1.29%)	30,751 (5.84%)	56,395 (4.21%)
Oceania	3,259 (0.70%)	13 (0.22%)	1,119 (0.47%)	138 (0.39%)	2,408 (0.46%)	4,251 (0.32%)
Unknown/stateless	947 (0.19%)	1,080 (18.27%)	4,845 (2.05%)	26 (0.07%)	0 (0.00%)	751 (0.06%)

For the primary analysis, one inclusion criterion was the ability to follow dietary changes in different groups, and also to compare immigrant groups with indigenous populations, with results being easy to tabulate. Furthermore, another inclusion criterion was the possibility to link dietary changes to diseases/disorders or to risk factors for CVDs. The primary search thus included vitamin D deficiency, iron deficiency, and caries. A secondary analysis was performed on other topics, that is, overweight/obesity, dyslipidemia, hypertension, diabetes, CHD, and dietary habits among immigrants. In this analysis, results were shown as different measures, or with disparate results in different immigrant groups, which explains why tabulation of the results was difficult to judge. It was also possible to use less strict criteria, for example, comparison with indigenous populations, or outcomes in relation to dietary changes.

## Results

**Fig. 1:**
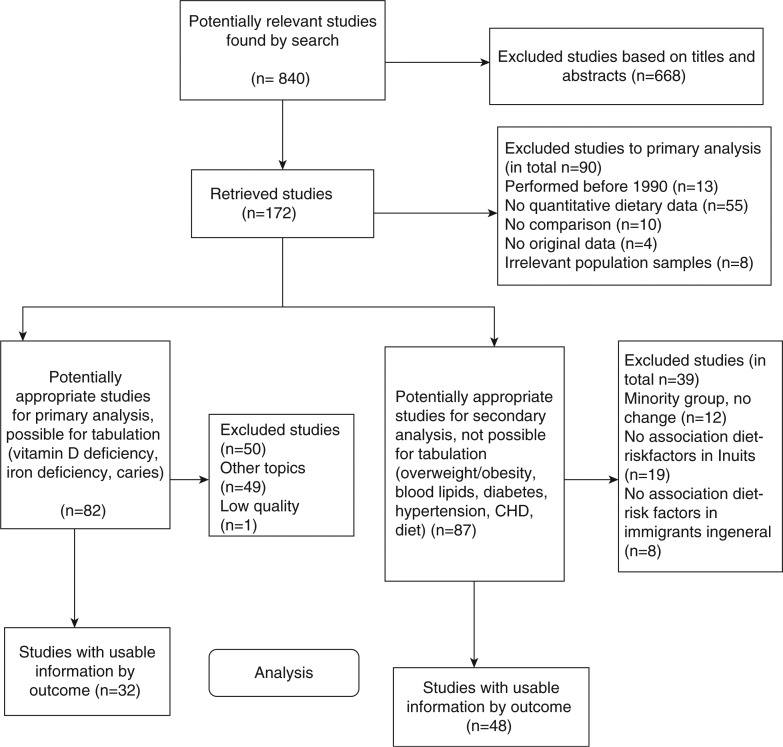
Flowchart for study of articles regarding population groups in dietary transition.

The search string is shown in the flow chart (Fig. 1). The total number of articles identified in 1990–2011 was 840, with 172 retrieved for reading in full text. For the primary analysis, 82 articles remained, and it was possible to extract data on relevant topics to tabulate in 32 articles. In a secondary analysis, less strict inclusion criteria were used, also including articles without distinct correlation between nutritional changes and resulting disorders. This analysis focused on overweight and obesity, also including analyses of dyslipidemia, hypertension, diabetes and CHD, and on dietary habits. A total of 48 articles were analyzed, with several articles addressing more than one area of interest. Articles of interest included fourteen on overweight and obesity or related measures; four on dyslipidemia; two review articles on diabetes, with twelve of the original articles found in the search included, and two further original articles on diabetes; three on blood pressure (BP) and/or hypertension; and seven on CHD. Moreover, nine articles included data on change or difference in food habits. The results from these studies could not be easily tabulated, and are instead summarized in the text, giving figures where possible and where of interest. Depending on the specific geographical setting and population sizes, dietary pattern differences were observed for population groups of Inuit (17–24) and Sami heritage in northern Norway (25–28), but changes in patterns over time could not be recorded. Also, factors other than dietary habits influence health outcomes (i.e. higher educational levels among Inuits in larger towns in Greenland and in Denmark; and psychosocial problems with alcohol abuse among Inuits in the small villages). No studies showed any distinct relation between change in dietary habits, increase in cardiovascular risk factors, or incidence of chronic disease (including diabetes) among the Inuit (29–47). There was no distinct relation between diet and cardiovascular risk factors and diseases in general among other immigrant groups (48–55).

### Studies on vitamin D

#### Vitamin D deficiency: classifications in reviewed studies

The included studies used different definitions of vitamin D deficiency, based either on levels of vitamin D or of parathyroid hormone (PTH), also using somewhat cutoff levels. In most studies, the most commonly used classification of vitamin D status according to levels of serum 25-hydroxyvitamin D (25(OH) vitamin D) or calcidiol was defined as: insufficiency (or mild deficiency) <50 and ≥25 nmol/L, moderate deficiency <25 and ≥12.5 nmol/L, and severe deficiency <12.5 nmol/L. However, in some studies, other terms or cutoff values were used: insufficiency as levels <37.5 and ≥20 nmol/ L (56); hypovitaminosis or deficiency as levels <50 nmol/L (57, 58); low levels as values <30 nmol/L (59–62); deficiency as values <20 nmol/L (56, 63–65), and severe deficiency as values <10 nmol/L (65, 66).

Secondary hyperparathyroidism was also used as a definition of severe vitamin D deficiency in many studies, mostly based upon blood levels of PTH, with PTH values >7.6 pmol/L (64, 65, 67) or >5.5 pmol/L (59–61) as most commonly used, with PTH >6.9 pmol/L in one study (56). Alternative definitions of hyperparathyroidism used values based on intact PTH (iPTH) with in one study the iPTH-level > 4.1 pmol/L (66), and in another study iPTH > 8.5 pmol/L together with 25(OH) vitamin D < 50 nmol/L, and s-Calcium ≤1.35 nmol/L (68).

Nutritional rickets was defined as both fulfilling biochemical inclusion criterion, that is, severe vitamin D deficiency (<12.5 nmol/L) or vitamin D deficiency (<25 nmol/L) and at least one other pathological biomarker (raised alkaline phosphatase, raised PTH, or low serum calcium), and clinical and radiological signs, including the effect of the given vitamin D treatment.

#### Vitamin D deficiency among children aged 0–16 years

Altogether, seven studies examining vitamin D deficiency among children were identified, five from Denmark (56, 67, 69–71), and two from Norway (72, 73). Also, one study from Sweden was found by snowballing (74).

High rates of vitamin D deficiency were found especially among immigrant girls aged 9–16 years (Table 2). Moreover, among the youngest children, that is, aged 0–4 years, the frequency of nutritional rickets was 50 times higher among children born in Denmark to immigrant parents compared to children born to Danish parents, although the frequency in the general Danish population was only 1‰.

**Table 2:** Vitamin D deficiency among immigrant children in the Nordic countries, with strength of evidence

Exposure/intervention	No. of participants (No. of studies)	Outcome variable (primary or secondary)	RR (95% CI)	Effect (95% CI)	No. of studies rated as A, B, or C	Strength of evidence	Comments
Vitamin D deficiency among children 0–16 years of age	309 (immigrants 191, controls 118) (3 studies)	Vitamin D deficiency (primary) Secondary hyperparathyroidism		Vitamin D deficiency (25(OH)D < 25 nmol/L): All 60% (46–74%) Secondary hyperparathyroidism (s-PTH > 7.6 pmol/L): All 26% (17–36%)	3 B	Convincing	Lack of comparative data with indigenous population, but high rate of deficiency	
Vitamin D deficiency among children 0–8 years of age	152 (immigrants 127, indigenous 25) (2 studies)	Vitamin D deficiency (primary) Secondary hyperparathyroidism Level of 25(OH)D, nmol/L		Vitamin D deficiency (25(OH)D < 25 nmol/L): All 46% (20–73%) Secondary hyperparathyroidism (s-PTH > 7.6 pmol/L): All 16% (–0.4 –30%) Level of 25(OH)D_2 + 3_: –13.7 nmol/L	2 B	Convincing	Lack of comparative data with indigenous population, but high rate of deficiency
Vitamin D deficiency among children 9–16 years of age	157 (immigrants 64, indigenous 93) (2 studies)	Vitamin D deficiency (primary) Secondary hyperparathyroidism Level of 25(OH)D, nmol/L		Deficiency (25(OH)D < 25 nmol/L): All 81% (63–100%) Secondary hyperparathyroidism (s–PTH >7.6 pmol/L): All 44% (31–58%) Boys 17% (4–29%) Girls 67% (43–91%) Level of 25(OH)D: Boys –13.7 nmol/L Girls –20.2 nmol/L	2 B	Convincing	Lack of comparative data with indigenous population, but high rate of deficiency
Nutritional rickets among children aged 0–16 years	198 (immigrants 167, indigenous 31) (3 studies)	Incidence of nutritional rickets		All immigrants born I Denmark 0.60 (0.39–0.81) ‰ per year Middle East immigrants born in Denmark 0.84 (0.48–1.23) ‰ per year	1A 1B 1C	Convincing	
Nutritional rickets among children aged 0–4 years	95 (immigrants 66, indigenous 29) (2 studies)	Incidence of nutritional rickets	49 (31–77)	All immigrants born in Denmark 0.97 (0.86–1.08) ‰ per year	1 A 1 B	Convincing	

#### Vitamin D deficiency among pregnant women

Altogether, five Norwegian studies of vitamin D deficiency among pregnant women were identified (59–62, 73).

Very high rates of vitamin D deficiency were found, with up to 15 times the risk of having subnormal levels of 25(OH) vitamin D levels compared to ethnic Norwegian women (Table 3).

**Table 3:** Vitamin D deficiency among immigrant pregnant women in the Nordic countries, with strength of evidence

Exposure/Intervention	No. of participants (No. of studies)	Outcome variable (primary or secondary)	RR (95% CI)	Effect (95% CI)	No. of studies rated as A, B orC	Strength of evidence (convincing, probable, limited-suggestive, limited-no conclusion)	Comments
Vitamin D deficiency pregnant women	*N*=227 Immigrant women *n*=227 Ethnic Norwegians *n*=61 (4 studies)	Vitamin D deficiency Severe vitamin D deficiency Vitamin D level Secondary hyperparathyroidism PTH level	Subnormal level (Calcidiol < 30 nmol/L): 2.78 (1.31–5.88) Low level (Calcidiol <20 nmolL): 6.90 (1.96–24.23) (25(OH)D_3_<30 nmol/L): 15.00 (5.13–43.83)	Difference in subnormal level (Calcidiol < 30 nmol/L): 62% (42–82%) Low level (Calcidiol <20 nmolL): 51% (30–72%) (25(OH)D_3_<30 nmol/L): 77% (62–92%) Difference in rate of secondary hyperparathyroidism (PTH > 5.5 pmol/L): 43% (25–61%) Difference in vitamin D level: S–25(OH)D_3_ –36 nmol/L (median) Difference in PTH level: 1 pmol/L (median)	4 B	Convincing	

#### Vitamin D deficiency among the general population

Ten studies of vitamin D deficiency among the general population were identified, two from Denmark (65, 66), seven from Norway (57, 63, 68, 75–78), and one from Sweden (58).

Very high rates of vitamin D deficiency were found among immigrant women from Arab countries, Pakistan, and Somalia (Table 4). High rates of vitamin D deficiency were found among immigrant men from Pakistan.

**Table 4:** Vitamin D deficiency among adult immigrants in the Nordic countries, with strength of evidence

Exposure/intervention	No. of participants (No. of studies)	Outcome variable (primary or secondary)	RR (95% CI)	Effect (95% CI)	No. of studies rated as A, B or C	Strength of evidence (convincing, probable, limited-suggestive, limited-no conclusion)	Comments
Vitamin D deficiency in the adult general population, women	*N*=2,279 ( immigrants *n*=1,104, indigenous *n*=1,064, country of origin *n*=111) (10 studies)	Vitamin D deficiency (primary) Severe vitamin D deficiency Secondary hyperparathyroidism Level of 25(OH)D, nmol/L Level of s-PTH pmol/L	Vitamin D deficiency (25(OH)D < 20 nmol/L), Arabs: 10.45 (4.63–23.58) Moderate deficiency (25(OH)D < 25 nmol/L), Pakistani: 177 (102–308) Moderate deficiency (25(OH)D < 25 nmol/L), Somali: spring 10.96 (3.44–34.95) Secondary hyperparathyroidism (s-iPTH ≥ 8.5 pmol/L + 25(OH) D < 50 + s-Ca ≤1.35 nmol/L), Pakistani: 3.86 (2.22–6.70)	Deficiency, low level: Calcidiol (< 20 nmol/L), Pakistani: 62% (43–81%) Moderate deficiency (25(OH)D < 25 nmol/L), Pakistani: 70.8% (63.3–78.4%) Moderate deficiency (25(OH)D < 25 nmol/L), Somali: autumn 77% (46–108%), spring 66% (34–99%) 25(OH)D (< 20 nmol/L), Arabs: 86% (56–116%) Severe deficiency (< 10 nmol/L), Arabs: 85% (76–94%) Severe deficiency (25(OH)D < 12.5 nmol/L), Pakistani: 20.7% (16.6–24.5%) Secondary hyperparathyroidism (s-iPTH ≥ 8.5 pmol/L + 25(OH) D < 50 + s-Ca ≤1.35 nmol/L), Pakistani: 17.3% (10.2–24.3%) Level Calcidiol, Pakistani: –43 nmol/L Level 25(OH)D, Arabs: –40.0 nmol/L Level 25(OH)D, Pakistani: –51.9; –50.1 nmol/L Difference in PTH level, Arabs: 2.0 pmol/L s-iPTH level, Pakistani: 2.6 pmol/L; 1.9, 2.43 (age-adjusted)	1 A 8 B 1 C	Convincing	
Vitamin D deficiency in the adult general population, men	*N*=1,697 (immigrants *n*=889, indigenous *n*=723, country of origin *n*=85) (7 studies)	Vitamin D deficiency (primary) Severe vitamin D deficiency Secondary hyperparathyroidism Level of 25(OH)D, nmol/L Level of s-PTH pmol/L	Secondary hyperparathyroidism (s-iPTH ≥ 8.5 pmol/L + 25(OH) D < 50 + s-Ca ≤1.35 nmol/L), Pakistani: 5.53 (2.82–10.84)	Moderate deficiency (25(OH)D < 25 nmol/L), Pakistani: 51.0% (43.7–58.2%) Severe deficiency (25(OH)D < 12.5 nmol/L), Pakistani: 8.7% (5.7–11.6%) Secondary hyperparathyroidism (s-iPTH ≥ 8.5 pmol/L + 25(OH) D < 50 + s-Ca ≤1.35 nmol/L), Pakistani: 13.4% (8.1–18.7%) Level 25(OH)D, Pakistani: –44.4; –44.7 nmol/L s-iPTH level, Pakistani: 1.2 pmol/L,; 0.8, 1.03 (age-adjusted)	1 A 5 B 1 C	Convincing	
Vitamin D deficiency in the adult general population, women and men	*N*=3,976 (immigrants *n*=1,993, indigenous *n*=1,787, country of origin *n*=196) (10 studies)			25(OH)D: –49.8 (–53.4–46.2) nmol/L	1 A 8 B 1 C	Convincing	
Vitamin D deficiency in the adult general population, women, compared to country of origin	*n*=209 Tamils in Norway *n*=89 Tamils in Sri Lanka *n*=111 (one study)	Vitamin D deficiency (primary) Level of 25(OH)D, nmol/L	Vitamin D deficiency (25(OH)D < 20 nmol/L), Tamils in Norway vs. in Sri Lanka: 5.50 (2.67–11.33)	Deficiency (25(OH)D < 25 nmol/L), Tamils in Norway vs. in Sri Lanka: 28.4% (16.4–40.4%) 25(OH)D: –17.1 (–21.0–13.2) nmol/L	1 A	Convincing	Only one study, but of high quality with distinct results
Vitamin D deficiency in the adult general population, men, compared to country of origin	*n*=229 Tamils in Norway *n*=144 Tamils in Sri Lanka *n*=85 (1 study)	Vitamin D deficiency (primary) Level of 25(OH)D, nmol/L		Deficiency (25(OH)D < 25 nmol/L), Tamils in Norway vs. in Sri Lanka: 31.9% (19.9–44.0%) 25(OH)D: –30.6 (–35.4–25.8) nmol/L	1 A	Convincing	Only one study, but of high quality with distinct results
Vitamin D deficiency in the adult general population, women and men, compared to country of origin	*n*=438 Tamils in Norway *n*=242 Tamils in Sri Lanka *n*=196 (1 study)		Vitamin D deficiency (25(OH)D < 20 nmol/L), Tamils in Norway vs. in Sri Lanka: 9.26 (4.91–17.44)	Deficiency (25(OH)D < 25 nmol/L), Tamils in Norway vs. in Sri Lanka: 29.5% (21.1–37.9%) 25(OH)D: –22.7 nmol/L	1 A	Convincing	Only one study, but of high quality with distinct results

### Studies on iron deficiency

Anemia due to iron deficiency was defined in a study on children as Hb < 11 g/dl and ferritin < 15 µg/L (79), and in a study on pregnant women, anemia was defined as Hb < 11 g/dl, and low ferritin level as values <12 µg/L (80).

The study on pregnant immigrant women indicates a higher risk of developing iron deficiency compared to indigenous women (Table 5), while studies on immigrant children showed no definite results.

**Table 5:** Iron deficiency among immigrants in the Nordic countries

Exposure/intervention	No. of participants (No. of studies)	Outcome variable (primary or secondary)	RR (95% CI)	Effect (95% CI)	Nuo. of studies rated as A, B or C	Strength of evidence (convincing, probable, limited-suggestive, limited-no conclusion)	Comments
Iron deficiency, children	*n*=74 (immigrants *n*=39) (1 study)	Anemia (primary) Sideropen anemia: Hb < 11 g/100 ml+ s-Ferritin <15 µg/L Empty iron deposits (ferritin < 10 µg/L) Severely low iron deposits (ferritin < 15 µg/L)	Anemia (Hb < 11 g/100 ml): 2.03 (0.54–7.65) Severely low iron deposits (ferritin < 15 µg/L): 2.14 (0.77–5.93)	Anemia (Hb < 11 g/100 ml): 9.1% (–7.9–26.1%) Sideropen anemia (Hb < 11 g/100 ml+ s-Ferritin <15 µg/L): 10.8% (–0.4–22.0%) Empty iron deposits (ferritin < 10 µg/L): 13.5% (1.0–26.1%) Severely low iron deposits (ferritin < 15 µg/L): 17.3% (–5.8 –40.4%)	1 B	Limited – suggestive	
Iron deficiency, pregnant women	*n*=76 (Pakistani women *n*=38) (1 study)	Anemia (primary) Low iron deposits (low ferritin) Microcytosis (low MCV)	Anemia (Hb < 11 g/100 ml): 1.63 (0.68–3.89) Low iron deposits (ferritin <12 µg/L): 4.33 (1.92–9.77) Microcytosis (MCV <82): 6.75 (2.73–16.70)	Anemia (Hb < 11 g/100 ml): 13.2% (–10.5–36.8%) Low iron deposits (ferritin <12 µg/L): 52.6% (23.5–81.8%) Microcytosis (MCV <82): 60.5% (31.8–89.2%)	1 B	Probable	

Besides these studies, one study excluded in the table only included children at one hospital with severe anemia due to iron deficiency, defined as Hb < 9 g/dl and ferritin < 15 µg/L (81). In addition, one study on pregnant Pakistani women (*n*=66) compared to Norwegian women (*n*=71) found that anemia (without specifying type of anemia or hemoglobin level) was more common among Pakistanis (OR 10.2; 95% CI 3.3–31.4) (82).

### Studies on dental caries among children

Altogether, seven articles of dental caries among children were found, one Danish (83), and six Swedish (84–89).

High rates of caries were seen among the younger immigrant children (i.e. <7 years, or the pre-school age in the Nordic countries) (Table 6), and among children of early school age (i.e. 7 years), while no conclusion could be drawn for teenagers aged 15 years.

**Table 6:** Caries among immigrant children in the Nordic countries

Exposure/intervention	No. of participants (No. of studies)	Outcome variable (primary or secondary)	RR (95% CI)	Effect	No. of studies rated as A, B or C	Strength of evidence (convincing, probable, limited-suggestive, limited-no conclusion)	Comments
Preschool children (< 7 years of age)	*n*=2,571 (immigrants 1,273, indigenous 1,298) (6 studies)	Manifest caries (primary) Dmf score	2.5 years: RR 2.46 (1.34–4.51) Adjusted OR 4.10 (2.06–6.14) From 1 to 2.5 years: Adjusted OR 2.34 (1.33–4.13) 3. 5 years: RR 3.13 (2.26–4.33) From 2.5 to 3.5 years: Adjusted OR 2.25 (1.36–3.73) 4 years, 2006: RR 1.83 (1.11–3.02)	2.5 years: 5.1% (1.7–8.6%) 3.5 years: 28.7% (20.5–36.8%) 4 years, 2006: 27% (5–49%) 4 years, dmfs: 1997: 2.2 (–0.6–5.0) 2002: 5.3 (2.3–8.3) 2006: 3.1 (1.1–5.0)	1 A 5 B	Convincing	
Early school age (7 years of age)	*n*=192 (immigrants 152, indigenous 40) (1 study)	Manifest caries Dmf score	dmfs + DMFS (mean caries experience with significance level according to Student's *t*-test): Turkish: RR 2.4** Pakistani: RR 1.9* Albanian: RR 4.1 *** Somali: RR 0.7 Arabian: 3.2*** PP (prevalence proportion rate): All immigrants: RR 1.48 (0.93–2.35) OR 3.14 (1.53–6.47)	PP (prevalence proportion rate): Turkish: 32% (–4 – 69) Pakistani: 21% (–14 – 56) Albanian: 32% (–9 – 72) Somali: 9% (–33 – 50) Arabian: 26% (–12 – 63) All immigrants: 25% (–5 – 55)	1 B	Probable	
Teenagers (15 years of age)	*n*=278 (immigrants 170, indigenous 108) (2 studies)	Manifest caries DMF score	DMFS (mean caries experience): Turkish: RR 1.9 Pakistani: RR 1.0 Albanian: RR 2.7*** Somali: RR 0.5 Arabian: RR 1.5 (differences according to Student's *t*-test) PP (prevalence proportion rate): All immigrants: RR 0.79 (0.65–0.96) OR 0.42 (0.18–0.96)	PP (prevalence proportion rate): Turkish: –4% (–22 – 14) Pakistani: –24% (–46–3) Albanian: 7% (–12 – 27) Somali: –48% (–81 – 15) Arabian: –2% (–22 – 18) All immigrants: –17% (–32–2)	2 B	Limited – no conclusion	

* denotes p<0.05

** p<0.01 and

*** p<0.001.

### Studies on overweight and obesity

Overweight was in general defined as body mass index (BMI) 25–29.9 kg/m^2^, and obesity as BMI ≥ 30 kg/m^2^. However, one study used the recommended Asian-specific cutoff for high health risk, that is, BMI≥ 27.5 kg/m^2^ (90). Recommended cutoff for waist circumference is 80 cm for women and 94 cm for men. Altogether, 14 articles were found on overweight or obesity, including four Norwegian, nine Swedish, and a Danish one.

One Norwegian study examined children aged 4 years and found that 26% of the immigrant children (*n*=33) had a weight equal to or above the 90th percentile compared to 6% of the Norwegian children (*n*=37) (48).

A Norwegian study on Pakistani women aged 25–63 years (*n*=198) found a mean BMI of 29.6 kg/m^2^, with a prevalence of overweight (BMI > 25 kg/m^2^) of 80%, and a rate above the Asian-specific cutoff for high health risk (BMI≥27.5 kg/m^2^) of 61% (90). Another study of Pakistani women showed a mean BMI of 29.5 kg/m^2^, and a mean waist circumference of 96.0 cm (91). In total, 40% were regarded as obese, and 95% had a waist circumference ≥80 cm (91). Yet another study used data from the Oslo Immigrant Health Study (Immigrant-HUBRO) performed in 2002 on immigrants from Pakistan or Sri Lanka aged 30–60 years (*n*=629), including a main questionnaire with food frequency questions, and an additional, more detailed questionnaire with questions related to life conditions as immigrants (52). BMI was positively associated with those of Pakistani origin, and inversely correlated with eating more hot meals, while waist–hip ratio (WHR) was positively associated with a dietary pattern with high-fat foods, and inversely associated with a degree of integration.

Altogether, nine Swedish studies on obesity were found, using data from different sources. Two studies were based on the Swedish Annual Level-of-Living Survey (SALLS) Immigrant Survey in 1996 on immigrants aged 27–60 years (*n*=1,957) from Poland, Chile, Turkey, and Iran. The first of these compared the immigrant groups with Swedish-born subjects from SALLS 1996–97 (*n*=2,975) and found a higher BMI in men from Poland and Chile, 0.77 kg/m^2^ (95% CI 0.23–1.31) and 0.72 kg/m^2^ (95% CI 0.29–1.15), respectively, and in women from Chile and Turkey, 1.88 kg/m^2^ (95% CI 1.40–2.36) and 1.49 kg/m^2^ (95% CI 0.92–2.07), respectively, when adjusting for education, physical activity, and smoking habits, using Swedish men and women, respectively, as reference groups (92). The second study used data both from SALLS Immigrant Survey 1996 and from the SALLS 1996–2002 on subjects aged 27–60 years (*n*=24,196), and found higher rates of obesity (BMI ≥ 30 kg/m^2^) among men from Turkey and Chile, and among women from Southern Europe, Turkey, and Chile (93).

Four regional Swedish studies were found. Two were based on data from Stockholm County, the first on 60-year-old men and women (*N*=4,228) in 1997–99, with data on Swedish-born subjects (*n*=3,329), and immigrants from Finland (*n*=327), OECD countries (*n*=335), and non-European countries (*n*=115). Among men, a higher mean BMI was found in the non-European group (28.1 kg/m^2^ vs. 26.8 kg/m^2^) as compared to the Swedish-born group, and among women, the non-European group showed a higher mean BMI (30.8 kg/m^2^ vs. 26.4 kg/m^2^) and waist circumference (94.5 cm vs. 86.0 cm) (54). Another study (94) on residents from two deprived neighborhood areas in a town in the southern part of Stockholm County (aged 18–65 years; *n*=289) found a higher risk of obesity (BMI ≥ 30 kg/m^2^) among immigrants of European origin (OR 2.79; 95% CI 1.06–7.36) and those of Middle Eastern origin (OR 3.10; 95% CI 1.51–6.40). A third study (i.e. the Swedish MONICA study of cardiovascular risk factors) was performed in Gothenburg on inhabitants aged 25–64 years (*N*=1,618), and an increased BMI and waist circumference was found among female immigrants (95). The fourth study (the Malmö Diet and Cancer Study) was performed in 1991–96 on 27,808 adults aged 45–73 years, including 3,110 European immigrants, and concluded that obesity was 40% more prevalent in non-Swedish Europeans compared to Swedes (51). Controlling for age, height, smoking, physical activity, and occupation, it was found that women born in the former Yugoslavia, southern Europe, Hungary, and Finland had a significantly higher percentage of body fat, and those from Hungary, Poland, and Germany had more centralized adiposity compared to Swedish women. Men born in the former Yugoslavia, Hungary, and Denmark had a significantly higher mean percentage of body fat compared to Swedish-born men, whereas Yugoslavian, Finnish, and German men differed significantly in mean WHR. Length of residence in Sweden was inversely associated with central adiposity in immigrants.

Three Swedish studies have been performed on specific immigrant groups. The first of these analyzed data on women from Iran (*n*=71), Turkey (*n*=36), or Sweden (*n*=50) (55) and found that Turkish-born women had higher BMI (28.4 kg/m^2^ vs. 24.7 kg/m^2^) and waist circumference (86.3 cm vs. 80.3 cm) compared to Swedish-born women. Another study was performed on immigrants from Turkey (*n*=238), aged ≥20 years, and it was found that BMI was higher among migrant men as compared to those in the area of origin in Turkey, 27.0 kg/m^2^ vs. 25.6 kg/m^2^, but not significantly so among women, 29.1 kg/m^2^ vs. 28.1 kg/m^2^ (96). A third study compared female Bosnian refugees aged 18–59 years (*n*=98) to Swedish-born women (*n*=95) and found a higher BMI among Bosnian women aged 42–59 years, 1.85 kg/m^2^, when adjusting for age, sex, and relationship between measured and self-reported BMI, using Swedish women aged 18–41 years as a reference group (97).

In a study on Inuit migrants in Denmark versus Inuits in Greenland, migrant women showed lower BMI and waist circumference than Inuits in Greenland, after adjusting for age, smoking, diet, and alcohol, 25.5 kg/m^2^ vs. 26.5 kg/m^2^ (*p*=0.02) and 83.3 cm vs. 88.3 cm (*p*<0.001), respectively (34).

Overall, the included articles show a risk of overweight and obesity in many, but not all, immigrant groups, especially among immigrants from non-Western countries.

### Studies on blood lipids

Blood lipid disturbances have been analyzed in three Swedish studies, and in a study of Inuit immigrants in Denmark. The first study investigated 60-year-old men and women (*N*=4,228) in Stockholm County in 1997–99, with data on Swedish-born subjects (*n*=3,329), and three immigrant groups, that is, from Finland (*n*=327), OECD countries (*n*=335), and non-European countries (*n*=115) (54). Risk of showing dyslipidemic values (HDL-cholesterol <1.03 for men and <1.30 for women) was higher in the non-European group as compared to the Swedish-born group (OR 2.06; 95% CI 1.35–3.159) when adjusting for sex, anthropometric values, hypertension, diabetes, lipid-lowering drugs, smoking habits, alcohol intake, socioeconomic factors, and diet. For high triglycerides (1.7 mmol/L), risk of showing this was only found in the sex-adjusted model for Finns (OR 1.31; 95% CI 1.01–1.71) and non-European immigrants (OR 1.98; 95% CI 1.34–2.93), and for apoB/apoA-I ratio (>0.90 for men and >0.80 for women) for Finns (OR 1.29; 95% CI 1.00–1.66) and non-European immigrants (OR 1.57; 95% CI 1.06–2.33). Concerning more specific immigrant groups, one study compared female Bosnian refugees aged 18–59 years (*n*=98) to Swedish-born women (*n*=95) and found higher values of triglycerides in Bosnian women aged 42–59 years (+0.92 mmol/L) and lower values of HDL-cholesterol in Bosnian women aged 18–41 years (−0.22 mmol/L) and 42–59 years (−0.26 mmol/L), when using Swedish women aged 18–41 years as reference group (97). Another study compared women from Iran (*n*=71), Turkey (*n*=36), and Sweden (*n*=50) (55) and found higher triglycerides (Iranians 1.39, Turkish 1.37 vs. 0.96 among Swedes) and lower HDL-cholesterol (Iranians 1.32, Turkish 1.33 vs. 1.68 among Swedes) among immigrant women.

A Danish study on Inuit migrants in Denmark and Inuits in Greenland (*n*=2,311) found that migrant men and women showed higher triglyceride levels compared to Inuits in Greenland in the general linear model when adjusting for BMI, age, diet, and other lifestyle factors, 1.66 mmol/L versus 1.21 mmol/L (*p*=0.01) and 1.33 mmol/L versus 1.10 mmol/L (*p*<0.0001), respectively, and migrant women showed higher levels of HDL-cholesterol (1.73 mmol/L vs. 1.56 mmol/L; *p*<0.001) (33).

In general, results show trends toward higher triglycerides and lower HDL-cholesterol among immigrants.

### Studies on diabetes

Two review articles were found, one on diabetes prevalence in general among immigrants, also including twelve of the original articles found in the search, and one on gestational diabetes. Another two original articles, not included in the reviews, were also found.

A higher diabetes prevalence was found in immigrants from non-European countries according to the general review citing 17 articles (15). Excess risks were found among the following immigrant groups compared to indigenous populations in the Nordic countries, with relative risks (RRs) shown in immigrants from the Middle East, 1.0–3.7 among men, and 2.2–7.8 among women; and in immigrants from South Asia, 2.4–5.5 among men, and 2.3–11.7 among women. Diabetes during pregnancy in immigrant women compared to indigenous women showed RRs of 2.3–7 according to the mentioned review (15), and were 5–10 times higher among women from South Asia in another review (98). A Swedish study on immigrants from Turkey (*n*=238, ≥20 years) found higher prevalence of diabetes in general (11.8% vs. 7.1%), and of diabetes among women (12.8% vs. 7.6%) and of impaired glucose tolerance (IGT) among men (17.8% vs. 4.9%) compared to subjects in the area of origin (*n*=1,549) (96). Among Inuit immigrants, the prevalence of diabetes (10.2%) and IGT (10.9%) was higher than ethnic Danes but not significantly higher than in the population of origin (99).

In general, a high risk of diabetes among immigrants from the Middle East and South Asia was found.

### Studies on BP and hypertension

A total of three related articles were found. One Swedish study on hypertension in 60-year-old men and women (*n*=4,228) found a higher risk among immigrants from Finland when adjusting for metabolic, lifestyle, and socioeconomic factors (adjusted OR 2.02; 95% CI 1.56–2.61), but a lower risk among immigrants from non-European countries (adjusted OR 0.52; 95% 0.34–0.80) (100). Also, in another Swedish study (*n*=24,196) based on data from the SALLS and the SALLS Immigrant Survey, a higher prevalence of hypertension was found in women from Finland (OR 1.83; 95% 1.25–2.67) compared to ethnic Swedes (93).

A Danish study on Inuit migrants in Denmark versus Inuits in Greenland (*n*=2,311) found systolic BP to be higher in migrants after adjusting for BMI, age, diet, and life-style factors (males 129.8 mm Hg vs. 121.9 mm Hg, females 124.4 mm Hg vs. 116.6 mm Hg) (41).

In general, high BP was only found in Finnish immigrants to Sweden, and Inuit immigrants in Denmark, while the risk seemed to be lower among non-Western immigrants.

### Studies on CHD

CHD incidence in immigrants has been studied using national or regional data in six studies (95, 101–105), and CHD prevalence in one twin study on Finnish immigrants (106). The first study included data on subjects aged 35–64 years from national Swedish registers (*n*=3,353,832, including 467,983 foreign-born) and found significantly higher RRs [adjusted for age, and socioeconomic status (SES)] in 10 out of 12 male (including European and other OECD countries, as well a Middle East and Asian countries) and six out of 12 female (including Finland, Poland, Bosnia and Middle East countries) studied immigrant groups (101). Another national study compared incidence rates over time in subjects aged 35–74 years between 1991 and 93 (*n*=4,042,690, including 459,125 foreign-born subjects) and between 1997 and 99 (*n*=4,152,620, including 561,991 foreign-born subjects), and found that CHD incidence decreased among men from Sweden, Finland, and OECD countries, RRs 0.88–0.90, while it increased in women from Sweden, RR 1.03, Southern Europe, RR 1.29, Iran, RR 1.40, and Turkey, RR 1.52 (102). The third national Swedish study used data on subjects aged 25–69 years between 1987 and 2001 and found that ‘first-generation immigrants from Finland, central European countries, other eastern European countries and Turkey had higher rates of CHD than men or women in the reference group. First-generation immigrant women born in southern Europe, other western European countries and Baltic countries had lower CHD risks than the reference group. Sons of both male and female first-generation immigrants showed CHD risks similar to or slightly higher than those of their parents. Amongst second-generation women, only subjects with Finnish fathers or mothers had higher risks of developing CHD than the reference’ (105). A fourth national Swedish study compared CHD mortality in immigrants aged 45–74 years in Sweden to CHD mortality in the country of origin, also using WHO data. Lower incidence density ratios were found for male immigrants from Norway, Finland, Germany, and Hungary, and for female immigrants from Germany and Hungary, and higher incidence ratios were found among male immigrants from Southern Europe as compared to in their country of origin (103).

Among regional Swedish Study, the MONICA study in Gothenburg (*n*=1,618), found an equal incidence of myocardial infarction among immigrants and Swedes (95), and a study from Stockholm County of persons aged 30–74 years (follow-up period 1977–96) found an increased risk for acute myocardial infarction among male and female immigrants (RRs 1.17 and 1.15, respectively) with an increased incidence primarily in subjects born in Finland, other Nordic countries, Poland, Turkey, Syria, and South Asia in both genders, from the Netherlands among men and from Iraq among women. The difference was still present after having spent more than 20 years in Sweden (104).

In the Finnish Twin Cohort study (*n*=1,534), it was found that Finnish migrant twins in Sweden showed a reduced prevalence of CHD compared to non-migrating twins (106).

The risk of CHD was found to be high in many different immigrant groups in Sweden.

### Studies on dietary patterns (and nutrient intakes) among immigrants

Altogether 10 articles on dietary patterns and nutrient intakes were identified. A Norwegian study was performed in 1994–95 among 4-year-old children with different parentage (i.e. two foreign-born parents *n*=33, two Norwegian parents *n*=37, one foreign-born and one Norwegian parent *n*=10, unknown status of parents *n*=2). In immigrant children, 41% received candy at least twice a week compared to 5% of the Norwegian children (48). Another Norwegian study among adolescents (aged 15–16 years) reported different dietary patterns across subgroups. The consumption of fruit and vegetables was high in the Middle East/North Africa group compared to the sub-Saharan African and the Indian subcontinent groups, but a higher consumption of sweets/chocolates and soft drinks/cola daily was seen among the African and Asian immigrant groups compared to adolescents of Western countries of origin (107).

Adult immigrants have been examined in two Norwegian studies. The first was an interview study of 25 Pakistani immigrant women, recruited through the Oslo Health Study 2000–2001.These women reported several changes in meal patterns, meal composition, and intake of different foods after migration to Norway (108). The second one, the Oslo Immigrant Health Study, used data on immigrants aged 30–60 years from Sri Lanka and Pakistan (*n*=629), including a main food frequency questionnaire (FFQ) supplemented by a more detailed FFQ on specific dietary habits in these groups (53). A majority of the Sri Lankans reported an increase in the consumption of meat, milk, butter margarine, and potatoes after settling in Norway. Around half of those from Pakistan reported increased consumption of oil, meat, fish, and potatoes. Multivariate regression showed that age was negatively related to increases in butter and margarine consumption, and a good command of the Norwegian language reduced the likelihood of increased consumption of oil and butter. The likelihood of having fat- and sugar-rich food patterns were reduced with age and years of education, whereas scoring high on an index of integration increased the likelihood of a fat-rich food pattern.

Four Swedish studies on dietary habits were found. The first study compared 60-year-old men and women (*N*=4,228) in Stockholm County in 1997–99 with immigrants from Finland (*n*=327), OECD countries (*n*=335), and non-European countries (*n*=115), using Swedish-born subjects as a reference group (*n*=3,329). Results from a FFQ indicated different dietary patterns regarding intake of non-oily fish, with lowest intake among non-European immigrants, of bacon and sausages, with lowest intake among men in the non-European group, and of fried potatoes, with highest intake among women in the non-European group (54). Another Swedish study was based on focus-group interviews with 20 female refugees from Bosnia-Herzegovina in 1993 (109). For breast-feeding, a period of 6–8 months was most common in this group of immigrants. Otherwise the ‘data analysis identified a large consumption of bread as staple food, with meat, vegetables, milk, cheese, legumes, egg and fish as complements. Self-sufficiency was noted with milk souring, jam making and the production of sweet fruit drinks. Homemade cheese and drying or smoking of meat were common methods of food storage’. Another Swedish study among women (*n*=157) from Iran (*n*=71), Turkey (*n*=36), and native Swedes (*n*=50), based on a standardized 24-dietary recall within a one-year sampling period, found that the immigrant groups had lower mean energy-adjusted intakes of magnesium and selenium than the Swedish group, whereas the mean intake of α-tocopherol, ascorbic acid, and folate was higher (55). The fourth Swedish study was performed on same-sexed twin pairs born in Finland (<75 years), with at least one twin migrating to Sweden (*N*=1,083 twin pairs). Dietary habits were assessed by a FFQ (response rate 71%), and in a subgroup of 76 male twin pairs also by a dietary history covering the previous year (49). Migrants in Sweden had a lower intake of typical Finnish foods like dark rye bread and berries, and an increased consumption of fresh food compared to co-twins living in Finland. The migrants consumed less potatoes and more rice and pasta. Migrants consumed sweet pastries less often and they also tended to cut visible fat off meat but on the other hand added salt to dishes.

Two Danish studies were identified on dietary habits. The first was based on the National Health Interview Survey 2005, including 136 non-western immigrants with Danish citizenship and 9,091 ethnic Danes aged 25–64 years. Odds ratios were higher among immigrants for reporting daily consumption of boiled vegetables (OR 2.50, 95% 1.77–3.53) and daily consumption of salad/raw vegetables (OR 2.84, 95% 2.02–3.99) (50). In the second study, Inuit migrants to Denmark were compared with Inuits in Greenland (*n*= 2,311). Migrants were more often found to consume fresh fruit daily (67% vs. 30%) and seal and fish less often than weekly (47% vs. 72%) compared to Inuits living in Greenland (41).

In general, the food patterns among immigrants were heterogeneous; to some extent adopting non-favorable Western food habits (i.e. with more sugar-rich food and beverages, and fat-rich food), but also to some degree preserving dietary habits rich in fruit and vegetables.

## Discussion

Among the main findings, prominent is the high rate of vitamin D deficiency among immigrants of all ages, especially among girls and women, including pregnant women. Other findings of importance include a high rate of caries among the younger immigrant children, and higher rates of overweight and obesity in different immigrant groups, with consequent risk of both diabetes and CHD. In contrast to this, the dietary patterns and changes among the immigrants studied seem to be ambiguous, with some being favorable, and others unfavorable.

The findings of a higher risk of vitamin D deficiency were consistent among non-Western immigrants of all ages, with extremely low serum levels of vitamin D and high levels of deficiency, also including secondary parathyroidism. In general, the vitamin D status seems to be satisfactory in the majority of the indigenous populations in the Nordic countries, with higher vitamin D levels in northern Europe compared to southern Europe (12), despite the lack of sunshine-induced vitamin D production in the body during the winter season. The reason for this paradox could be explained by other factors than the intensity of sun light. Nutritional factors, including food fortification and supplement use of vitamin D, are probably of importance. For instance, the mean vitamin D intake in Sweden is calculated to be twice that of other European countries (12). Besides, in Norway the use of cod liver oil is especially common, and the risk of vitamin D deficiency is inversely associated with this as well as with the intake of fatty fish (75). However, fish-eating dietary habits and vitamin D supplement use are not widespread among non-Western immigrants in the Nordic countries. Other factors of importance are skin color and sun-exposing behavior. The indigenous populations in the Nordic countries have a lighter skin color and also a sun-exposing behavior, thus enhancing vitamin D formation by sun light. This is supported by a Danish study finding a gradient in vitamin D levels from women with darker skin and a sun-avoiding behavior to women with lighter skin and a sun-exposing behavior (65), and a Norwegian study where 97% of Pakistani women covered their bodies, except for hands and face, when outside (62).

Only one study was found comparing the vitamin D status of an immigrant group in Norway with the situation in the country of origin, in this case Sri Lanka, showing a higher risk of vitamin D deficiency in Norway (78). In a review of Turkish, Moroccan, Indian, and sub-Sahara African populations in Europe and their countries of origin, the vitamin D status was concluded to be low compared to indigenous European populations, while the vitamin D status in the countries of origin was highly variable (110).

The high rate of vitamin D deficiency among pregnant women is serious, since vitamin deficiency is associated with a higher risk of complications during pregnancy and delivery (111), and also could affect fetal growth (61) and contribute to rickets among children (112).

Despite low levels of vitamin D, some studies found no difference in biochemical markers of osteoporosis between immigrants and indigenous populations (63, 64, 68). When present, differences between immigrants and indigenous populations were modest, for example, with somewhat higher bone alkaline phosphatase among Pakistani immigrants in Norway compared to ethnic Norwegians, despite a large difference in the rate of secondary hyperparathyroidism (77). Another study found a highers-Calcium among the Pakistani immigrants (76).

As only a few studies analyzed the possible risk of iron deficiency, it is hard to draw any conclusions regarding that. There is certainly a need for more studies on this topic. Among the youngest, immigrant children started consuming infant formula and cow's milk earlier during the first year of life and had a more frequent use of sweet beverages than the Norwegian children (79, 81). For pregnant immigrant women, it was indicated that higher parity, less common use of iron supplementation, and greater use of the traditional Pakistani diet (chapatti and other whole-wheat meal bread) was the reason for the higher risk of iron deficiency in Pakistani women compared to Norwegian women (80).

The higher caries risk among the younger immigrant children is also alarming. The reason for this seems to be a more frequent use of sugary beverages, especially at night, a larger number of meals per day, and a higher intake of candy (83–85, 88).

Dietary patterns and changes among immigrants are essential within the topic of this review, but the findings were somewhat contradictory. In the classical migration study among Japanese–Americans, it was concluded that the group most acculturated to Western culture had three- to five-fold excess in CHD prevalence (113). An increased rate of overweight and obesity is observed in migrant populations in general (9), and this seems true also for many immigrant groups in the Nordic countries. Nutritional changes are important but other factors also contribute to overweight and obesity, that is, sedentary lifestyle, and the urbanization and mechanization found in society. Many jobs in the past required heavy physical labor, and travelling was on foot or bicycle, and thus mechanization and automation, including the increasing use of motor vehicles, could largely explain the historical trend toward indolence and obesity (114). The increased intake of sugar-rich products, often referred to as the coca colonization (115), is of importance not only as a risk factor for caries as pointed out but also for overweight and obesity. Adopting less healthy dietary patterns, and relinquishing healthy dietary patterns, such as higher intake of fiber-rich products in the form of legumes and wholegrain products are both negative parts of the acculturation process. A whole-wheat meal could also contribute to iron deficiency among Pakistani women. However, knowledge seems to be important, as higher education is associated with healthier dietary and life-style habits, and dietary interventions leading to higher knowledge have shown good results (90). Thus, it is important to facilitate the process of integration–acculturation of immigrants. Food patterns have changed in Nordic countries during the past decades, not only with the increased intake of sugar-rich products but also with positive influences from different immigrant groups. These changes have led to greater variety in the Nordic cooking practices, and to trends using traditional products from the Nordic countries, such as different kinds of berries and different kinds of grains, including rye, oats, barley, and spelt. This could mean that we are able to take the best from two worlds, that is, integrating the indigenous inhabitants into the immigrants’ cultures, as a good and mutual integration.

A recent review of dietary changes in immigrants of South Asian origin in Europe concluded that even if the picture of dietary changes is complex, some factors seem important in explaining the increased risk of obesity, type 2 diabetes, and CVDs (116). A main dietary trend is an increase in energy and fat intake, and a decrease in carbohydrate intake, with a switch from wholegrains to refined carbohydrates, and as a consequence low fiber intakes.

However, there are also other factors influencing the development of obesity. Because of genetic variation (117), some population groups may be more vulnerable when exposed to over-nutrition. Low SES and place of residence are also of importance (118), with some immigrant groups having low SES (119).

In accordance with the higher obesity risk in immigrants, the prevalence of diabetes is higher in immigrants of non-European origin, with women from South Asia being the most affected group. Hypertension seems to be less common in non-Western immigrants, while blood lipids tend to be disturbed with high triglycerides and low HDL-cholesterol. CHD risk in general seems to be increased in many immigrant groups, but in addition to over-nutrition and obesity, other factors such as smoking habits may play an important role. However, migrating from a high-risk country in this respect might lower the risk compared to the population in the country of origin. One other factor that may be of importance is vitamin D deficiency. Vitamin D deficiency is regarded as a risk factor for many diseases, including diabetes mellitus and CVD but also many types of cancers and infectious diseases. The optimum level of vitamin D is not established, but there is evidence that a concentration of >50 nmol/l could be optimal (120).

There are some limitations to this study. The literature search was performed on one occasion, with no further search according to reference lists from recovered articles of interest. Only one individual reviewed the abstracts, although with some assistance to avoid missing articles of possible interest. The focus was on areas of clinical interest, where important results were found, and some areas with less conclusive results, or more complicated associations between dietary changes and outcomes, were not further explored. Strengths were that the search was broad, yielding many articles of possible interest, where the most important areas of interest actually were covered.

The quality of included studies varies, with many studies of high quality on vitamin D deficiency, and also with several studies of fairly high quality on dental caries among children, with consistent and convincing results. Most studies on CHD used regional or national register, and were of high quality, with rather consistent results. Studies on overweight, obesity, and diabetes were of varying quality, also including larger studies of high quality, and the general results with higher risk among non-European immigrants seem well substantiated. For blood lipids, dyslipidemia, BP, and hypertension, there is a dearth of studies, and from those studies found, it is difficult to generalize, as they are of varying quality and also with a low number of participants. Besides, one problem that became apparent was the lower attendance rate in some immigrant groups and in some studies. Thus, there could be a risk of biased results, especially for iron deficiency, blood lipids, dyslipidemia, BP, and hypertension. Even if some studies included in the overview of overweight, obesity, and diabetes were small, the results still are consistent for the larger immigrant groups. The studies on dietary patterns occupies a special position, and are somewhat difficult to interpret, with heterogenic and to some extent conflicting results. Problems with low participation rate and also with registering of food habits may also bias the results, which should be interpreted with some caution.

## Conclusion

The main conclusion is the very high rate of vitamin D deficiency among immigrants, with special regard to pregnant women, children, and adolescents, which calls for intensified attention and active measures. The high rate of caries among immigrant children, especially among pre-school children, calls for more active measures. Another concern is iron deficiency among pregnant immigrant women, and to some extent immigrant children, where more research is needed. The risk of overweight and obesity seems to be high in many immigrant groups, and consequently the risk of diabetes and CVDs, especially CHD are also high.
